# Information Distribution in Multi-Robot Systems: Generic, Utility-Aware Optimization Middleware

**DOI:** 10.3389/frobt.2021.685105

**Published:** 2021-07-27

**Authors:** Michał Barciś, Agata Barciś, Nikolaos Tsiogkas, Hermann Hellwagner

**Affiliations:** ^1^ Karl Popper Kolleg on Networked Autonomous Aerial Vehicles (KPK NAV), University of Klagenfurt, Klagenfurt, Austria; ^2^ Department of Mechanical Engineering, Division RAM, KU Leuven, Leuven, Belgium; ^3^ FlandersMake@KULeuven, Core Lab ROB, Leuven, Belgium

**Keywords:** multi-robot systems, information distribution, adaptive communication, information utility, communication optimization, Monte Carlo tree search

## Abstract

This work addresses the problem of *what* information is worth sending in a multi-robot system under generic constraints, e.g., limited throughput or energy. Our decision method is based on Monte Carlo Tree Search. It is designed as a transparent middleware that can be integrated into existing systems to optimize communication among robots. Furthermore, we introduce techniques to reduce the decision space of this problem to further improve the performance. We evaluate our approach using a simulation study and demonstrate its feasibility in a real-world environment by realizing a proof of concept in ROS 2 on mobile robots.

## 1 Introduction

Advances in the field of *multi-robot systems* (MRS) have allowed sophisticated real-world applications in science and industry. Some examples include underwater archaeology (Allotta et al., 2015), search and rescue (Yazdani et al., 2019), and manufacturing (Hoshino et al., 2008).

The information distribution problem refers to the decision process of *what* information to exchange, *when*, and *with whom* to achieve a good performance of the whole system while obeying the limited resource utilization, such as available throughput or energy usage. Despite the need to exchange a multitude of information in MRS, often the literature neglects the role of communication in such systems. Usually, it is either taken for granted or the main focus is on maintaining the connectivity (Amigoni et al., 2019). Only recently, the problem of information distribution in MRS began to receive attention (Marcotte et al., 2019; Best et al., 2019; Fowler et al., 2020; Anderson and Hollinger, 2021).

In the problem of information distribution, the decision process aims to optimize the communication in MRS with specified constraints. To perform such an optimization, the importance of each communication event has to be assessed. Usually, the importance of these events depends on the mission currently executed by the MRS. Hence, domain knowledge about the mission has to be incorporated into the solution. This can be achieved in a direct or an indirect way.

A *direct* approach (Marcotte et al., 2019; Best et al., 2019; Unhelkar and Shah, 2016; Roth et al., 2006; Anderson and Hollinger, 2021) simulates the course of the mission to estimate the impact of each communication event on the mission objective. Although such approaches guarantee the best performance for a specific application, they assume the ability to forward-simulate the mission, which might require considerable amount of effort or be infeasible.

On the contrary, *indirect* approaches assign a utility to the data being processed during the mission. They communicate the most valuable (i.e., providing the highest utility) pieces of information, assuming this would positively impact the mission performance. A common way to define such utilities is given by information-theoretic measures such as KL-divergence (Fowler et al., 2020; Williamson et al., 2009). However, such approaches imply that the application logic takes into account information uncertainty. In many practical applications that is not the case, for example, often outdated information is just used as if it were current. Therefore, it might be challenging to integrate these solutions into existing systems. Another indirect approach is to design utility functions tailored to a specific application (Kassir et al., 2015; Becker et al., 2009; Mazdin et al., 2020). Often, such approaches treat information in a myopic manner, i.e., taking into account only the immediate value of communication without evaluating its long-term impact. More sophisticated models are rarely considered (Kassir et al., 2015; Becker et al., 2009; Szer and Charpillet, 2004). In this work, we present a solution based on an indirect approach.

Another aspect to consider when speaking about the information distribution problem is how to define what information can be exchanged. It is commonly assumed that the whole state of an agent (e.g., a robot) can be shared during a single communication event (Best et al., 2019; Unhelkar and Shah, 2016; Williamson et al., 2009). This approach allows researchers to avoid the question of *what* to communicate and focus only on the question of *when* to communicate. Although in many cases it is fair to assume that all information could be transmitted in a single message, it is not applicable in a generic setting (Fowler et al., 2020). As an example, suppose the state of an agent consists of its position, battery level, and camera images. In that case, it might be enough to regularly send only the first two components and reduce the rate of resource-hungry transmission of images.

Such a simplification results in a significant reduction of the decision space, which enables efficient computations (Best et al., 2019). In this paper we avoid this assumption, while still benefiting from a similar reduction of the decision space in specific situations (see Section 3.2.2 for details). To achieve this, we categorize all information exchanged among agents into *information types*. It is an abstract way to group together information that possesses similar properties (e.g., only the most recent information of a given type is important for a robot). For instance, an information type could be the position of a robot, the camera image or the map generated by all robots collaboratively.

In this work, we focus on the question of *what* to communicate. Although this question is extensively studied in the context of specific applications, there is still a need for generic methods addressing it (Tsiogkas and Lane, 2019). Therefore, we exploit an indirect approach as a way to decouple the information distribution optimization process from the mission at hand. This allows us to propose a generic information distribution optimization middleware that is easily integrable into any robotic system communicating by exchanging messages, without any modifications to the application logic.

**FIGURE 1:**
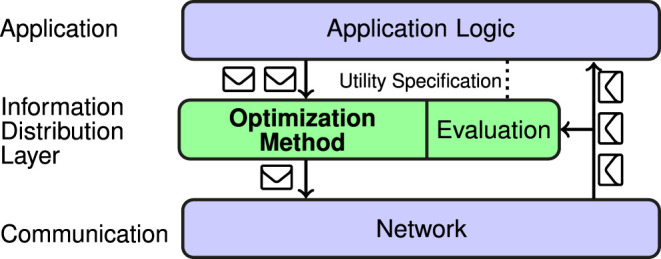
Design of the proposed information distribution middleware. Application logic generates messages that are then intercepted by the introduced information distribution layer. The optimization method decides which ones are worth forwarding to the communication layer (i.e., sending). In order to do so, it utilizes the evaluation model, which can be adjusted for the mission at hand using utility specifications.

Our solution consists of two blocks, marked with green color in Figure 1. The first block is a generic optimization method that can be transparently incorporated into any application. The second one is an evaluation model that defines the utility of information. An evaluation model taking into account the specifics of a mission at hand can bring application-awareness into the information distribution middleware.

To the best of our knowledge, this is the first work that presents an information distribution optimization method which holds all of the following three properties: it is transparently integrable into existing systems; it jointly considers exchange of various information types; and it is able to work in real-time (online) on robots. The main contributions of this work are as follows.

• Design of an online and transparent information distribution middleware that comprehensively (for all information types) optimizes information exchange in an MRS taking into account non-myopic, mission-aware message utilities (Section 2.1).
• Introduction of two techniques to reduce the decision space of the proposed method, thus facilitating its use in practical applications (Section 3.2).
• Method for mission characterization based on the difficulty of the information distribution optimization problem in a given mission (Section 4.1).
• Proof of concept implemented in Robot Operating System 2 (ROS 2) on mobile robots and a simulation study performed using a network simulator (ns-3) (Section 4).

## 2 Problem Formulation

In this work, we focus on deciding which messages should be sent to maximize the obtained utility, constrained by the limited available communication resources, such as energy or throughput. Formally, such an objective can be expressed as the following optimization problem to be solved collaboratively by the MRS:

$$\begin{array}{l} \overset{\text{maximize}}{M_{\text{rcv}}\subseteq ℳ}U\left(M_{\text{rcv}}\right)\\ \text{subject}\;\text{to}\;\text{network}\;\text{constraints }\left(\text{eq}.\;\left(5\right)\right), \end{array}$$ (1)

where $M_{\text{rcv}}$ is a set of messages received by any agent, ℳ is a set of all messages that can be generated, and U is an evaluation model that returns a utility value. The evaluation model U is an important part of this problem. In practice, many formulations of U employ compromises to allow for efficient methods to solve the maximization problem. However, in this work, we allow for any definition of U, i.e., we assume it can assign any value to any subset of messages. Such a formulation of the problem has a complexity growing exponentially with the number of messages (Pynadath and Tambe, 2002). The messages can influence each other in arbitrary ways, thus all possible subsets need to be considered. Moreover, as the number of messages is usually high and the decisions need to be made fast, looking for an optimal solution is infeasible. Hence, most research efforts tackling this problem (including this paper) are focused on identifying heuristics that are applicable in practice (Roth et al., 2006).

### 2.1 System Design

The system setup considered in this work consists of multiple agents (e.g., robots) exchanging messages to pursue a common objective. The agents broadcast messages over a single wireless channel giving them knowledge of all communication in the system. It is assumed that agents are working within each other’s communication range, though in principle multi-hop connections could also be used. We assume an efficient channel access method that guarantees a fair usage of the communication medium and resolves potential message collisions.

This setup is motivated by a realistic application of MRS where multiple (between 5 and 10) mobile robots collaboratively execute a mission. The robots are connected using an IEEE 802.11 wireless network in an ad-hoc mode. Examples of such applications are presented in our previous works (Barciś et al.,; Barciś and Bettstetter, 2020; Mazdin et al., 2020; Mazdin and Rinner, 2021; Rinner et al., 2021). All of these works are motivated by a use case of a group of UAVs collaboratively performing a 3D reconstruction of an unknown area.

We design the solution as a transparent middleware that can be integrated into existing systems. A diagram of the design is presented in Figure 1. The solution could be perceived as an additional layer introduced between the application logic and the network. It consists of two parts: the optimization method, which is the main contribution of this work, and an evaluation model. The evaluation model is aware of all messages exchanged in the network, but it does not modify them in any way. In principle, a very generic model can be used, possibly completely independent of the mission at hand. The model introduced in our previous work (Barciś et al., 2020) is designed specifically to be used in this setup. It allows the optimization method to make mission-aware decisions and is able to consider both myopic and long-term impact of a message (see Section 2.2 for details).

All messages are handled as follows. First, a message is generated by the application logic. Second, it is processed by the information distribution middleware deciding to either drop it or forward it to the network layer (i.e., send it), which finally transmits it to the other agents. The objective of the information distribution middleware is to make this decision in such a way that the received utility, as defined by an evaluation model, is maximized, taking into account the constraints.

Our approach requires the knowledge of the generation time of all messages (also the ones that are yet to be generated in the future). Often reliable mission-specific estimations for these times can be designed. For instance, an agent could be sending a message whenever it enters a warehouse or its battery is low. In this work, we estimate the generation time by assuming the messages are generated periodically. This assumption often holds in practical situations. For example, robots generate sensor data, such as their position, with a predefined, fixed rate. As soon as the first position message is received, we can estimate the generation times for all the future position messages.

### 2.2 Utility-Based Evaluation Model

An integral part of the introduced information distribution middleware is the *evaluation model*. It should be able to assign a numerical value (utility) to any set of exchanged messages. Although the optimization method introduced in this work does not depend on a specific evaluation, its design is inspired by the evaluation model introduced in our previous work (Barciś et al., 2020). This model is also utilized in our implementation in Section 4. In the following, we will briefly describe the essentials of the model. For a more detailed description and motivation, refer to the publication introducing the model (Barciś et al., 2020).

The evaluation model introduces the term *total utility* to denote the resulting numerical value assigned to a set of all exchanged messages. It is defined based on *message utility functions*. The message utility functions depend on the information type and describe how the utility of a message of this type changes throughout the mission. To compute a numerical value from such a message utility function, it needs to be integrated over the total mission time. Finally, *utility aggregation functions* define how to aggregate (i.e., combine) utilities from multiple messages over time. This approach allows us to consider messages in a non-myopic manner, i.e., taking into account their usage during the whole mission. It also enables us to express how messages influence each other. For instance, it is possible to state that a message is not valuable if previously some other message was received.

An interesting characteristic of this evaluation model is that the total utility is known only when the mission is finished. After all, often it is impossible to predict when and how the message will be useful throughout a mission. Thus, in order to use the model for information distribution optimization during the mission, properties of each exchanged message (e.g., reception time, message content, etc.) have to be estimated. Some properties, for instance, message reception time, can be estimated based on the network performance and these are incorporated into our implementation. However, sometimes the utility is based on mission-dependent message properties, like the position of the robot at the time the message was generated. These properties cannot be reliably estimated in a generic setting. Providing a mission-specific estimator for such properties improves the performance of the whole system. Often even a very simple estimator shows good performance. An example is given in Section 4.3, which states that in this specific mission an image will probably contain the same amount of red pixels as the previous image generated by the same agent.

## 3 Methods

We propose a method to tackle the information distribution optimization problem based on Monte Carlo Tree Search (MCTS) (Coulom, 2006; Kocsis and Szepesvári, 2006). MCTS is a heuristic decision algorithm based on the Monte Carlo method. It is exceptionally valued in the field of game theory (Corera, 2016), but has also been successfully applied in other fields (Pepels et al., 2014), including the information distribution problem (Best et al., 2018). The algorithm enables an efficient traversal of a decision tree, considering more promising options first. It executes a pre-selected number of iterations (also called play-outs or roll-outs). The higher the number of iterations, the better is the result, eventually leading to the optimal solution. This allows us to easily balance the exploration depth (thus better optimization results) and computation time needed for each decision. This ability was the main reason why we have decided to utilize this method. Furthermore, the fact that MCTS eventually explores the whole tree is also beneficial for the problem of information distribution. Other heuristic approaches sometimes discard parts of the solution space as not worth exploring, which in general might result in a sub-optimal solution even with unlimited computational resources. For readers not familiar with the MCTS algorithm, we provide its detailed description in Supplementary Appendix A.

### 3.1 Information Distribution Optimization With Monte Carlo Tree Search

Each time an agent needs to make a decision whether it should send a message or not, it builds a new MCTS tree. All generated messages from all agents are incorporated into this tree. Hence, the agent takes into account other agents, which allows it to make decisions optimized for the whole system. The information about messages generated by other agents is not always available and has to be estimated (for instance, in the case of future messages). In this work the estimation is done using the assumption about periodic messages (see Section 2.1) and their expected utilities are estimated using mission-specific estimators (see Section 2.2 with examples in Section 4.3). A similar approach would also work for other setups where message generation times are known or can be reliably estimated.

Each node at the *i*th level of the tree represents a decision: whether the *i*th message should be sent or dropped. To take into account the messages that might still be in transfer, we construct the decision tree including also messages generated in the past. The maximum age of messages to consider is based on the current network latency.

**FIGURE 2:**
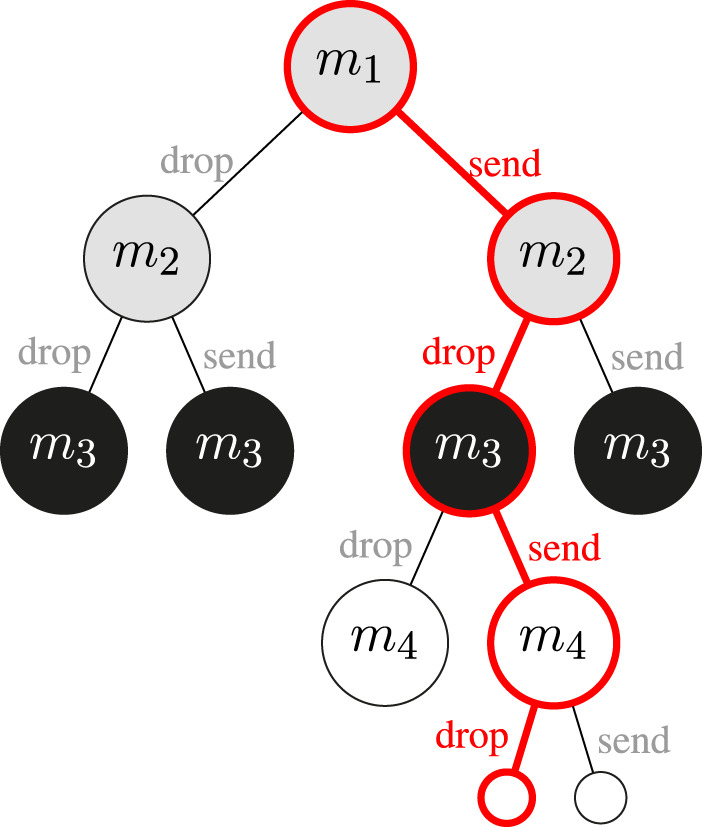
Example of a tree constructed by MCTS. The black nodes represent the message to decide about. Grey nodes represent messages generated in the past that might have not been received yet. White nodes represent future messages.

An example of a decision tree obtained using MCTS is visualized in Figure 2. For the sake of the example, we assume that each message is generated by a different agent. In order to make the example easy to follow, it consists of only four messages and shows a result of a relatively low number of iterations of the MCTS algorithm. This particular tree is constructed in order to decide if message $m_{3}$ should be sent or not. In practice the tree would have been explored much further into the future. However, even in such a simplified setup it is possible to observe the asymmetrical growth of the tree. It occurs because the MCTS explores more promising options first. The red outline marks the path that is the output of the algorithm. The decisions represented by this path are likely to provide the highest utility. Based on it the agent would choose to send message $m_{3}$.

Even though the MCTS provides decisions for all considered messages, we use it only to decide about the one just generated. There are multiple reasons for this design choice. First, before the next message is generated, the agent’s knowledge might change. This could influence the estimated utility values and hence invalidate the previous decisions. Second, a message considered in the tree might be generated by another agent, which is able to better estimate its value.

The key design decision in an MCTS-based method is how to assign rewards for final states and how to calculate scores of intermittent states. Typically, after each tree expansion step, the MCTS algorithm simulates the whole execution of the decision process with random future decisions. However, in our problem, this is infeasible. Thus, we utilize another common approach: instead of performing the MCTS simulation step, we score the states using a scoring function. The scoring function is a problem-specific heuristic that provides a simple way to determine which states are better than others. We decided to formulate it based on the ratio between the value of utility in the given state and a maximum value of utility, achieved by receiving all messages. Specifically, we use the following scoring function:

$$S\left(v\right)=\frac{U\left(M_{v}\right)}{\psi U\left(ℳ\right)},$$ (2)

where *S* is a function assigning a score to a node *v* of the decision tree, U is the evaluation model returning the utility of exchanged messages, $M_{v}$ is the set of messages exchanged up to node *v*, ℳ is the set of all messages that could ever be transmitted during the mission and ψ is a value representing the progress of the mission. In our experiments, we set $\psi =\frac{t}{t_{\text{end}}}$, where *t* is the generation time of the message considered in *v* and $t_{\text{end}}$ is the duration of the mission. In the simulation-based study (Section 4.2) the value of U(ℳ) is known, but in the practical experiments (Section 4.3) we estimate it.

For now, we did not explicitly consider any network constraints. Without this, MCTS could decide to transmit all messages. An interesting way to include the network constraints would be to make them a part of U. Such a U could utilize a network simulator (e.g., ns-3) in order to estimate the expected value of sending the given subset of messages and, for instance, taking into account message drop rate and delays that could occur in a network. Assuming a reliable network simulator, the solution would take into account that sending too many messages results in potential loss and latency increase, which in turn decreases utility. Even though we find such solution interesting, it might be infeasible given the overhead associated with network simulation. Hence, in our implementation we estimate the future latency as an average observed latency and use the technique described in Section 3.2.1 to introduce network constraints.

An inherent part of such decision problems is a balance between exploration and exploitation^1^. To give some intuition, in our problem an exploitation-only solution would be a greedy method that sends a message whenever it increases the utility, whereas an exploration-only approach would prioritize trying previously untested decisions. To maintain a balance between these two extremes in MCTS, often the UCT formula (Kocsis and Szepesvári, 2006) is used to decide which node to go to in the MCTS selection step (cf. Supplementary Appendix A.1.1). Always the node with the highest UCT value is chosen. The UCT formula is:

$$\mathrm{UCT}\left(v\right)=\bar{S}+c\sqrt{\frac{\ln n_{p}}{n_{v}}},$$ (3)

where $\bar{S}$ is a mean score (Eq. 2) of all descendants of *v*, $n_{p}$ is the number of visits of the MCTS algorithm to the parent node of *v*, $n_{v}$ is the number of visits to node *v*, and *c* is called a discovery factor. High values of *c* result in a more exploratory behavior, whereas low values promote exploitation of the already constructed tree. In our experiments, we fix *c* to 0.35 in order to prioritize high-valued decisions (i.e., sending the message).

### 3.2 Reduction of Decision Space

In the following, we propose two methods to reduce the decision space with the aim of increasing the performance of the optimization middleware. The first method considers network constraints and is applicable to all information types, albeit providing only a small performance boost. The second method, on the other hand, is applicable only to specific types of information, but is able to reduce the tree growth from exponential to polynomial.

#### 3.2.1 Reduction Based on Network Constraints

So far, our approach considered any subset of messages to be sent, even if transmitting them would cause obvious overuse of resources (e.g., causing network congestion). Unfortunately, it is difficult to predict how the network will perform under such stress. In principle, this could be done using a network simulator. However, such an approach has two major problems. First, simulating transmission of many messages at each node of the decision tree requires considerable computational resources if an advanced simulator is used, whereas using simple simulations provides less reliable results. Second, in practice other processes (i.e., not considered by the information distribution middleware) might also run in the network and their behavior might be impossible to simulate (e.g., a system using aggressive retransmission schemes could cause congestive collapse of an overloaded network).

To address these problems we introduce the following reduction. In addition to avoiding resource overuse, it enables simple simulations to be used and at the same time reduces the decision space of the MCTS. At the core of the reduction lies the following predicate:

$$\forall t\in \left[0,t_{\text{end}}\right].\left|W\left(t,M_{\text{sent}}\right)\right|\leq A\left(t\right),$$ (4)

where $M_{\text{sent}}$ is the set of sent messages, *W* is the windowing function that returns a subset of all messages affecting resource utilization at time *t* (i.e., being transmitted at time *t*), operator |⋅| measures how much resource is used by a subset of messages and A(t) specifies how much resource is available at time *t*. Intuitively, such a predicate is false if and only if there exists a moment when the resource is overused. To give an example, if A(t) is a constraint specifying the number of messages that can be simultaneously transmitted, then the operator |⋅| is simply a power of subset returned by a windowing function. In such a case, the predicate is false if at any time *t* there are more messages being transmitted than is allowed by A(t).

However, in practice, evaluating such a predicate for each time instant is infeasible. Therefore, we introduce an additional assumption: a message consumes a constant amount of resources for the whole time of transmission, e.g., it is being transmitted with a constant speed. This allows us to reduce the predicate from Eq. 4 to the following one:

$$\forall t\in \left\{m.t_{sent}|m\in M_{sent}\right\}.\left|W\left(t,M_{sent}\right)\right|\leq A\left(t\right),$$ (5)

where $m.t_{\text{sent}}$ is the moment when *m* is sent. This predicate allows us to check the same condition but can be evaluated only in discrete moments, i.e., when a message is sent.

Such a predicate can be defined for a variety of resources, e.g., network throughput, or energy. For each tree node in the decision process, we check the truth values of such predicates. If any of them is false, we do not consider the node nor its descendants in the decision process. The additional computational complexity of this check depends on the specific definition of a predicate, but for many practical predicates (e.g., limited network throughput) it can be realized in amortized constant time^2^ [for details see our implementation (Barciś, 2020)]. In the following paragraphs we present analytical results summarizing the efficiency of the proposed constraint. The derivations of these results are provided in Supplementary Appendix B.

Many of such predicates can be simplified to a constraint that allows to send only less than *r* messages in a window. In such a case, the number of nodes at the *i*th level of the tree is equal to: $2^{i}-2^{i-k}\sum_{j=r}^{k}\left(\begin{array}{c} k\\ j \end{array}\right)-\left(2^{i-k}-1\right)\left(\begin{array}{c} k-1\\ r-1 \end{array}\right)$, where *k* is the number of messages generated in a window.

The decision space with such constraints still grows exponentially with the number of messages. If the resource is very limited, the reduction can be significant. However, when the resource is abundant, there is almost no impact on the size of the tree. To give some intuition: in the case when all messages can be sent, the constraint has no impact, i.e., the number of nodes stays the same and is equal to $2^{i}$. When $r\approx \frac{k}{2}$, the number of nodes is approximately $2^{i-1}$, so it allows us to consider one more message with the same computational effort. Finally, if only a few messages can be sent (specifically, r<<k), the number of tree nodes is reduced to below $2^{i-k}$, allowing us to consider up to *k* more messages with the same computational cost.

In our experiments, we use one such predicate for a *network throughput constraint*: we define *A* as a constant function returning fixed maximum network throughput, operator |⋅| as the sum of the sizes of all messages in a subset divided by the window length, and use the following windowing function:

$$W\left(t,M_{sent}\right)=\left\{m\in M_{sent}|t\leq m.t_{sent}\leq t+T\right\},$$ (6)

where *T* is a parameter defining the window length. Low values of *T* result in a constraint that does not allow many messages to be sent at similar times. High values of *T* provide fewer restrictions, but might result in a temporary resource overuse. For the experiments we have empirically set this value to T=0.5 s, since it provides a middle-ground between the two described extremes and works well in our experimental setup.

#### 3.2.2 Reduction for Markovian Information Types

Many information types used in MRS exhibit a Markovian property, i.e., only the most recent message of such a type is useful (brings utility). In this section, we present a reduction method that significantly limits the decision space for such information types.

Let us recall that the messages in the tree are ordered by generation time. We define the *state* of a node in the decision tree as a set $\left\{\tau_{p}|p\in P\right\}$, where P is the set of all information types and

$$\tau_{p}=\{\begin{array}{cc} m_{p,\text{last}} & \text{if }\;p\;\text{is}\;\text{Markovian},\\ M_{p} & \text{otherwise}, \end{array}$$ (7)

where $m_{p,\text{last}}$ is the most recently received message of type *p* and $M_{p}$ is the set of all messages of this type received so far.

If two nodes at the same level of the decision tree are sharing the same state^3^, we will call them *tantamount nodes*. We observe that any decision made in a descendant of one tantamount node has exactly the same influence on the mission as a corresponding decision in any other tantamount node. Consequently, the utilities achieved from these future decisions are also equal. Therefore, it is enough to consider a tantamount node that brings the highest utility, as the other ones will for sure not perform better. It means that all tantamount nodes with utility lower than the highest one do not have to be expanded anymore.

At each level of the decision tree there is a finite number of states equal to:

$$2^{\hat{n}}\prod_{p\in P_{M}}n_{p},$$ (8)

where $\hat{n}$ is a number of messages from non-Markovian information types, $P_{M}$ is the set of Markovian information types and $n_{p}$ is the number of messages of type *p* generated so far. For each state, all tantamount nodes are reduced to a single node. Thus, after this reduction the number of nodes in the tree grows exponentially only with respect to messages of non-Markovian information types. For Markovian information types it grows polynomially.

This observation can be incorporated into the MCTS algorithm by maintaining a set $\xi_{i}$ of the best nodes for all states at each level *i* of the decision tree. Each time the tree is expanded, the algorithm checks if the newly expanded node *v* is better than its tantamount node v′ in the set $\xi_{i}$ of the current level. If this condition holds, the descendants of v′ are attached to *v*, v′ is replaced by *v* in $\xi_{i}$, and all statistics of the tree are updated to accommodate this change. In a case where *v* achieves worse utility than v′, the expansion is canceled and not considered in the future.

This modification introduces only a small computation overhead of maintaining a set of states after each simulation of MCTS. To analyze computational cost we assume there are *n* messages in the tree. If, for the implementation of this specific set, a tree-based set is used, additional O(log(n)) operations per simulation are needed. Whereas, when using hash table, the expected number of additional operations is just O(1) and in the worst case it is O(n). Both of these solutions introduce a negligible cost in practice, because each simulation has to perform O(n) operations anyway. The overhead is small compared to the benefit of greatly reduced decision space.

## 4 Evaluation

In this section we present a twofold result. First, in a simulation study (Section 4.2) we show that the introduced method produces good results in a multitude of applications. Second, through a robotic proof of concept (Section 4.3), we present the applicability of the proposed method in a real-world scenario.

However, arguing about the performance of a solution to an information distribution problem is inherently complicated due to the multitude of applications and conditions to consider. For instance, a method adjusted for distributed photogrammetry might not work well for a multi-robot search and rescue mission. Nevertheless, state of the art publications addressing the problem of information distribution are usually ignoring this aspect and evaluating the solutions using specific applications (Marcotte et al., 2019; Best et al., 2019; Unhelkar and Shah, 2016; Williamson et al., 2009; Fowler et al., 2020; Roth et al., 2006). Hence, in Section 4.1 we propose a novel evaluation methodology that presents the performance of our solution in different scenarios. Then, we use it to present the rest of the results.

### 4.1 Evaluation Method

Instead of focusing on a specific application, we introduce a mission *characteristic* that intuitively describes the difficulty of the information distribution problem for that mission. Then, we present three scenarios with different characteristics and show how our approach performs in them.

**FIGURE 3:**
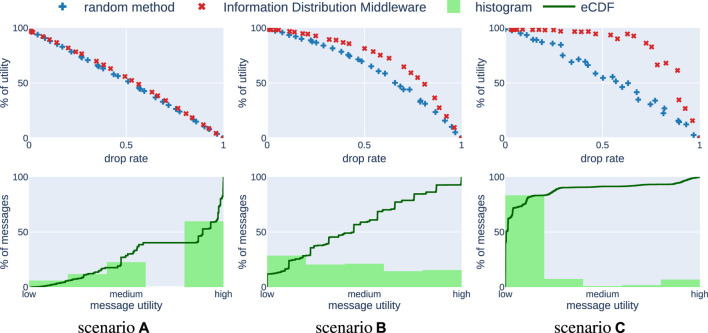
Results of comparison between the baseline random method and the Information Distribution Middleware in the simulation study. In scenario **(A)** most of the exchanged messages are of high utility. In scenario **(B)** there is a uniform distribution of messages with respect to utility. Finally, in scenario **(C)** most of the messages are of low utility. These plots can be reproduced by running the DropRateVsUtility experiment in our software framework (Barciś, 2020).

As a characteristic of a mission, we could use a distribution of utilities of all generated messages in the mission: what percentage of messages provided high utility, what low, etc. Such a characteristic would allow us to identify how important it is to optimize information distribution for a given application. However, sending one message influences the utility of other messages. Thus, it is not straightforward to obtain such statistics. For instance, in one execution of the mission a message could be very valuable, whereas in the other it could provide almost no utility, because the same information was shared using a different message. Hence, instead of using a distribution of utilities we approximate the distribution of expected utilities of all messages. In order to acquire the proposed statistics, we first perform experiments using an algorithm that randomly drops messages with different probabilities. Then, for each message exchanged during all experiments we compute how much utility it provided for the mission and plot the distribution of these values. The results in Figure 3 are obtained by running 30 simulations with different message drop rates, resulting in around 12,000 exchanged messages.

We consider three scenarios. In *scenario (a)* a majority of messages are very important for the mission, hence have a high utility value. An example of such a mission could be an application of unmanned aerial vehicles (UAVs) to map an unknown area and share with others map fragments that were not yet explored. Then, each map fragment is equally important and hence brings similarly high utility. In such a mission even if the information distribution optimization is realized perfectly, the benefit is very low, because even a random method would choose to send most of the valuable messages. On the other hand, in *scenario (c)* a majority of messages is not useful and there are just a few very important messages. For this case an example could be a search and rescue mission performed by UAVs, which are periodically sharing their status. For most of the time UAVs are just reporting the fact that they did not locate a victim. However, the messages stating that the victim has been found are much more valuable, but also relatively rare. In this scenario the main goal of the information distribution optimization is to find and deliver these important messages. For such applications it is fairly simple to showcase the superiority of some information distribution approach over a random scheme. Even a very simple solution that transmits only the high-valued messages would provide results much better than a random approach. Apart from these two extremes one can think about scenarios in between, with similar numbers of messages of different utility. We will refer to this middle-ground as *scenario (b)*. An example of such a scenario could include UAVs measuring pollution around a building with an assumption that measurements made closer to the building are more important. The characteristics of these three scenarios are depicted in the bottom row of Figure 3 as histograms. The expected utilities of messages are normalized w.r.t. the message with the highest utility value and characterized with adjectives “low”, “medium” and “high”. Additionally, the empirical cumulative distribution function (eCDF) for this data set is plotted with a green line.

### 4.2 Simulation

Each scenario is implemented and evaluated in the network simulator *ns-3*. We have configured the simulator to be as close as possible to our robotic setup, i.e., using an ad-hoc IEEE 802.11 b/g network with messages broadcast in a non-reliable manner using UDP. In all simulated scenarios there are 10 agents—this number is motivated by the practical applicability of systems of this size and similar to the number of robots we used for practical experiments (8, described in Section 4.3). Simulations with lower and higher (up to 16) number of agents were also conducted, but for the sake of compactness, are not presented in this publication. The longer the experiment, the more consistent the results. Hence, instead of performing multiple experiments and averaging results, for each set of parameters we run an experiment for a predefined time. We have empirically determined the mission duration of 100 s to provide consistent outcomes. The software is open source and available online to make the results reproducible (Barciś 2020).

Throughout the whole experiment, each agent generates with a frequency of 1 Hz a piece of information about itself, e.g., its battery level or position. The actual semantics of a message is not important, as we are pursuing an indirect approach and only consider the utility provided by an evaluation model. As evaluation model, we use the approach described in Section 2.2. Message utility functions are set to be linearly decreasing functions with variable slope and initial value. This allows us to specify for each message how long it generates utility, how much, and express the fact that the information could be overwritten by another message. They are not based on real-world information types. This definition allows us to introduce a variation between utilities of single messages and also to vary how messages influence each other.

The difference between three scenarios is introduced by splitting all messages into ten sets, numbered from 0 to 9. Then the utility provided by the message from *i*th set is multiplied by weight $\omega_{i}$. For scenario (a), $\omega_{i}=3^{i+1}$; for scenario (b), $\omega_{i}=1000i+1$; and for scenario (c), $\omega_{i}=i^{0.1}+1$.

The MCTS is performing 1,500 iterations for each decision, which in our implementation takes around 0.3 s using a personal laptop computer (Intel Core i7–7500U, 2.7 GHz). This value has been chosen to keep the duration of experiments manageable. Higher number of iterations provides better results, at the cost of longer decision time.

The results are presented in the top row of Figure 3. The *y* axis, labeled *% of utility* refers to the ratio between achieved utility and maximum possible utility, i.e., obtained by exchanging all generated messages without delays. We compare our optimization method (red ×) with a randomized one (blue +). Each sample is a result of a single 100 s long experiment. For each algorithm we ran 30 experiment runs. In the case of a random method each experiment is run with a different probability of dropping a message. For our method the number of messages to send is constrained using Eq. 5 and also varies with different experiments. The plotted value is based on the received messages, so the loss naturally occurring in the network is also taken into account.

We observe that our method performs very well in comparison to a method that randomly drops messages if there are many low-value messages (scenario (c)). It means that our approach is able to identify which messages bring high utility and sends them. On the other hand, in scenario (a), most of the messages bring high utility. It does not matter which ones are chosen, hence our algorithm cannot perform much better than the randomized method. Scenario (b) presents a middle-ground between the other two extremes.

### 4.3 Proof of Concept Using Mobile Robots

In contrast to deterministic simulations, in the real world the changing environment makes it very hard to reproduce similar experiments on robots in a reliable fashion. Hence, the aim of the proof of concept presented in this section is to show that the approach is applicable in practice on affordable hardware and to qualitatively confirm the results of the simulation-based study. We also utilize this opportunity to present how the layered design of our method enables fast and easy integration into existing systems.

The use case is prepared using eight *Pololu Balboa* robots modified for our previous work (Barciś et al., 2019). The platform allows us to easily achieve mobility and integrates a very popular computing platform, *Raspberry Pi 3B+* with *Raspberry Pi Camera Module V2*. It is set up to work in an IEEE 802.11 b/g ad-hoc network with multi-hop connection support provided by the *Babeld* protocol. The software is built in *Python* on top of a *Robot Operating System 2 Eloquent Elusor* (ROS 2) robotic framework with a default *eProsima Fast RTPS* implementation of the *Data Distribution Service* (DDS) standard. The robots’ real-time clocks are synchronized using *Chrony* with a root mean square (RMS) clock discrepancy offset below 0.1 ms.

By using an ad-hoc network together with ROS 2 we create a fully distributed system, i.e., without any single point of failure enabling robots to join and leave the mission freely.

The application is designed to simulate a surveillance mission. The robots move around and send a stream of 640×480 px images from their cameras to the operator’s computer. The aim of the system is to monitor red objects. This is a simplification made in order to clearly present our goals, but a similar system could additionally classify objects and look for some particular classes, e.g., cars. Alternatively, an infra-red camera could be used. Then, the red color could suggest presence of people or animals.

**FIGURE 4:**
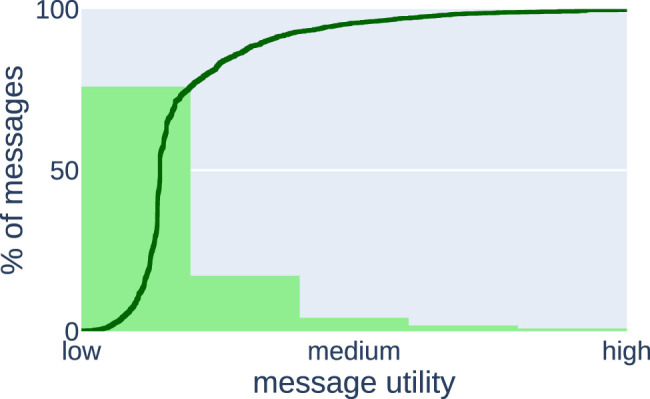
Characteristics of the proof of concept, which show that a random algorithm would not perform well in this setting as most of the messages are of low utility.

Figure 4 presents the characteristics of this mission obtained using data containing all generated messages from a single experiment and processed in the same way as in Section 4.1. We present it mainly to demonstrate that the introduced concept of characteristics can be used to analyze practical missions, but also to show that even in a simple application a randomly dropping algorithm would not perform well.

As a baseline, the system has been implemented purely in ROS 2 without the use of our information distribution middleware. The performance was mediocre. When pictures were sent rarely enough not to overflow the network, the refresh rate was low (around 1.5 Hz). Setting a higher refresh rate resulted in a network congestion. Even though we used a communication scheme without retransmissions (non-reliable setting of DDS), delays of image frames increased above 3 s and the variation of frame rates was high. This resulted in a situation where it was possible to completely miss a red object, even when it was positioned in front of a robot for a couple of seconds.

Then, we have incorporated our method in three simple steps: intercepting image messages sent by the original system, defining utility functions for them to be dependent on the amount of red pixels in the image, and specifying that an image is expected to have the same number of red pixels as the previous one. Specifically, we have used the following utility function:

$$U_{\text{m}}\left(m,t\right)=\{\begin{array}{cc} 0 & \text{if}\;t<m.t_{\text{rcv}}\\ U_{\text{vid}}\left(m,t\right)\left(1+\alpha \;m.\text{red}\right) & \text{otherwise} \end{array},$$ (9)

where *m* is a message, *t* is a moment at which we want to evaluate the utility function, $m.t_{\text{rcv}}$ is the message reception time, and m.red is a percentage of red pixels in the transmitted image. $U_{\text{vid}}$ is a utility of a message that ensures a good quality of experience taking into account human perception. It assumes that doubling the frame rate of image stream improves the experience of a person observing it by a constant factor. The exact definition of $U_{\text{vid}}$ is taken from the paper introducing the evaluation model (Barciś et al., 2020) (Eq. 28). Low values of parameter α prioritize frequent updates from each robot, whereas high values prioritize the images with red pixels. In the experiments α=10, which we determined empirically to provide a good viewing experience. The number of MCTS simulations needed to be tuned and set to 300, which resulted in a reasonable balance between low latency and good system performance.

**FIGURE 5:**
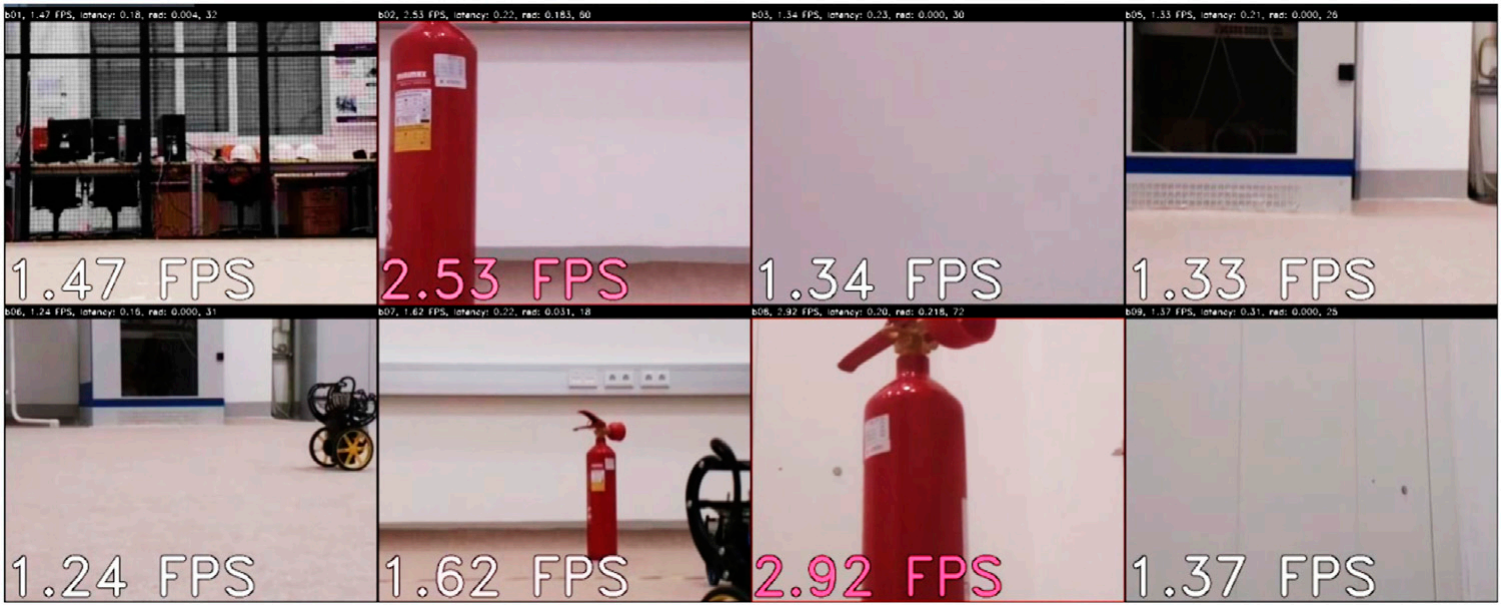
Screenshot of the demonstrator in which the robots are exchanging images and our method maintains a limited throughput constraint while giving preference to images containing more red pixels.

These modifications greatly increased the system usefulness. A screenshot of a running application is presented in Figure 5. It can be seen that a robot seeing a red fire extinguisher transmits images the most frequently. The robots that see some red pixels (e.g., wheels of other robots) are also sending information more often than other ones, but overall the link is shared fairly. As soon as a robot sees a red object, the frame rate of its video stream increases. We cannot show this in a picture, but it can be observed in a video attached to this publication. Note that this behavior emerges autonomously, no additional logic was programmed.

On the downside, our method increases latency of the system because of the added computational overhead. This overhead depends mainly on the number of simulations performed by MCTS. In the presented scenario, with MCTS performing 300 simulations, the latency caused by the optimization method was around 0.1 s for each message. Before the introduction of the middleware, when the network was not overloaded, images were received approximately 0.2 s after being generated. Afterward, the latency grew to approximately 0.3 s. For the presented scenario, this is not a big issue: if one is looking for a red object, it is better to see it with a delay than to miss it completely. Furthermore, the proof of concept was not programmed with performance as the main priority and makes use of inefficient technologies, e.g., the Python programming language.

Finally, the goal of the demonstrator is not to show this particular behavior. After all, a similar application could be realized in a more efficient way if it was designed specifically for this use case. We show that our method is applicable in practice and can easily transform a relatively simple application into a much more sophisticated one. Furthermore, the simulation-based study shows that the method provides similar benefits to more complex systems, for which designing a specific information distribution optimization scheme might be infeasible.

## 5 Conclusions and Outlook

We presented a generic method to optimize information distribution that can be integrated into any robotic system that exchanges messages. The proposed method allows the agents to select and exchange the most relevant information types, increasing the mission performance. In addition to the performance benefits, the proposed method is easy to integrate. The only step needed to benefit from it involves defining the utility functions for each used information type. The proposed method shows good performance in diverse scenarios and is demonstrated both in simulation and in experiments involving mobile robots.

We see multiple ways future work could be developed. First of all, there is certainly a need for a unified benchmark or data set in order to make comparisons with other approaches quantifiable. The mission characteristics presented in this work could be a first step toward a more general solution. Furthermore, the comparisons with state of the art are relatively hard, because usually no code is provided and the evaluation is done using hand-crafted applications. Hence, a standardized set of experiments could facilitate the future work and make similar research more reproducible.

In addition, the properties of different information types can be investigated and exploited to develop efficient methods for information distribution. Ideally, such methods could be then incorporated into a generic system to increase its performance for that given information type (similarly to the approach presented in this paper for Markovian information types).

Finally, automating the generation of utility specifications and estimators would minimize the user effort to integrate the proposed middleware into existing applications. One potential approach could be to utilize statistical/machine learning-based methods to autonomously determine utility specifications and obviate the need for any additional application-related modifications.

## Data Availability

The software developed and used in this study is available at http://github.com/zeroos/infdist.

## Footnotes

1 The concept refers to the situations in which an algorithm needs to decide to choose a solution that is known to provide good results (exploit) or test a new option to look for a better solution (explore).

2 This means that the check would on average require constant time, but sometimes might incur more computations. In our case, the first check requires a number of operations proportional to the size of the window and the next ones can be done in constant time.

3 For completeness, the state should also contain an information about the network constraint utilization. It is not included for the sake of simple presentation. In particular, the presented considerations hold without changes if the constraint from Eq. 5 is used with window size smaller than the publishing periods of Markovian message types.
