# Supplementary material for: Information Distribution in Multi-Robot Systems: Generic, Utility-Aware Optimization Middleware
Source: Front Robot AI. 2021 Jul 27;8:685105. doi: 10.3389/frobt.2021.685105 (PMC8353533; doi:10.3389/frobt.2021.685105)
Supplement: Supplementary file 1 [file DataSheet1.pdf]

Appendices

A MONTE CARLO TREE SEARCH

Monte Carlo tree search (MCTS) (Coulom 2006; Kocsis and Szepesvári 2006) is a heuristic decision algorithm based on Monte Carlo method. It is widely used in the design of artificial intelligence, for instance, in order to play games. In fact, the method was first introduced to play Go (Coulom 2006) and since then almost dominated the field (Cazenave et al. 2019). Perhaps the most remarkable program utilizing this method is *AlphaGo* developed by *DeepMind Technologies* (formerly *Google Deepmind*), which was the first computer program to beat a professional Go player (Corera 2016). The method shown its applicability also in other problems, like playing nondeterministic video games (Pepels et al. 2014), planning (Mansley et al. 2011), and the problem of information distribution in MRS (Best et al. 2018).

MCTS is based on classic Monte Carlo methods, where the object of study (e.g., a continuous field) is randomly sampled in order to estimate some properties of this object. In the case of MCTS the object of study is a decision process and it is being randomly sampled by traversing a tree where each node represents a decision.

A.1 Base of operation

The following description explains the generic base of operation of the basic version of MCTS algorithm. The description is intertwined with an example that presents how this algorithm can be used in order to solve the problem of information distribution in MRS.

The MCTS algorithm starts with a tree consisting of a single root node. This node represents the state where none of the decisions are made.

EXAMPLE. *Let us assume we have a set of* 4 *messages:* $\{m_1, m_2, m_3, m_4\}$ *and we need to decide which ones of them are worth sending. We need to make* 4 *decisions: for each message we should decide if it should be sent or dropped. This state is represented by the root node.*

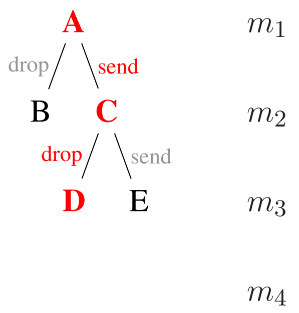

Figure 1. Example of the selection step. Node D was eventually selected, which represents a state in which message m_1 is sent, m_2 is dropped and the decision about the other messages is not yet made.

The method involves running a number of simulations. Each simulation involves testing a different set of options for each decision and examining the outcomes. The more simulations, the better the estimation.

Then, for each simulation the algorithm performs four steps: Selection, Expansion, Simulation, and Backpropagation. The steps are described in the following subsections and summarized in pseudocode in algorithm 1. Additionally in section A.2 we provide references to the Python source code used in our experiments.

A.1.1 Selection

We start from a root node and traverse the tree down until a leaf node is reached. A leaf node is a node without any children. For each node we choose the child to go to based on the UCT formula (given in Eq. 3). The selection step is described with pseudocode in line 8 of algorithm 1 and an example of this step is presented in fig. 1.

A.1.2 Expansion

If in the leaf node there are still some unresolved decisions (e.g., messages for which we did not decide if they should be sent or not), in the expansion step we choose one of them and create as many children as there are options for the considered decision. In principle the decision could be chosen at random, but usually some heuristic is utilized to first consider decisions that are more significant. In our implementations the decisions (i.e., messages) are ordered by generation time to assure deterministic outcomes.

Then, one of the newly created children is chosen randomly and considered in the next step. We will call this chosen child the *expanded child*.

The pseudocode for this step is provided in line 13 of algorithm 1.

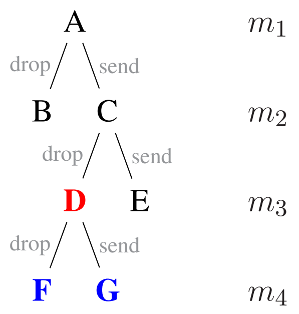

Figure 2. Example of the expansion step. Node D is being expanded and nodes F and G are the newly created children nodes.

EXAMPLE. *This example is visualized in fig. 2. Let us assume we are in a leaf node D. There are still two messages for which the decision was not made: m_3 and m_4 generated at, respectively, times 3 s and 4 s. Message m_3 is generated earlier, so we will consider options related to it. We can either send it or not. Hence, we create two children nodes — one representing a situation when m_3 is sent (G), the other when it is not (F). In both of these children the decision regarding message m_4 is still unknown.*

A.1.3 Simulation

The goal of this step is to estimate the value of the expanded child form the previous step. In classic MCTS at this point all of the unresolved decisions will be made randomly. Then, the resulting state would have to be evaluated which would result in a numerical value being assigned to it (for instance, 1 for a won game and 0 for a lost game).

However, often the set of decisions might be big and hence even performing them randomly might be computationally intensive. An alternative approach to this step is to asses the value of a given state without considering future decisions. This assessment could be done, for instance, by utilizing domain-specific expert knowledge or be based on machine learning.

This step is presented in line 21 of algorithm 1.

EXAMPLE. *Let us assume node F was chosen as the expanded child. We can utilize some evaluation method in order to assess the situation in which message m_1 is sent and messages m_2 and m_3 are dropped. The method could, for instance, assume that bigger messages are more useful. As a result a numerical score should be assigned to node F.*

The evaluation model utilized in our implementation is briefly summarized in Section 2.2 of the paper.

A.1.4 Backpropagation

Executing the simulation step provided a new information about the expanded node. This information needs to be propagated to all its ancestors in order to improve their estimated values. The ancestors of node F are marked in fig. 3 and the pseudocode of this procedure starts in line 23 of algorithm 1.

In order to make the final decision, we start from a root node and then always go to the child that was visited the most times. When the child that represents the decision we are interested in is reached, we have the result. This procedure is described with pseudocode in algorithm 2.

A.2 Implementation

In order to use MCTS in our experiments we have utilized a free and open-source Python implementation available on Github[1]. We have forked it and ported to Cython[2] in order to improve performance. Additionally, we changed the selection step to be deterministic in order to make our experiments reproducible.

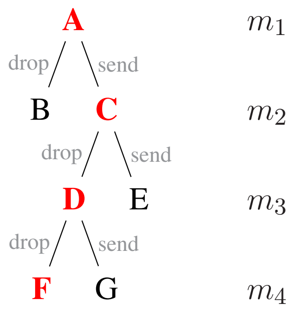

Figure 3. Example of the backpropagation step. All nodes that should be updated are marked with red color.

[1] https://github.com/ImparaAI/monte-carlo-tree-search
[2] https://github.com/zeroos/monte-carlo-tree-search

Algorithm 1 The MCTS algorithm. The main procedure starts in line 28.

```
1: class Node
2:     Node parent
3:     Node[] children
4:     bool expanded := False
5:     Decision[] unresolved_decisions
                                          ▷ e.g., a set of messages to decide on
6:     int visits := 0
7:     float value := 0

8: function SELECT(tree_root)
9:     Node node := tree_root
10:    while node.expanded do
11:        node := child of node with the highest UCT score
12:    return node

13: function EXPAND(node)
14:    decision := choose one of the unresolved decisions in node
15:    option := choose one of the options for decision
                                          ▷ e.g., drop a message
16:    expanded_child := create a child representing option
17:    node.children.APPEND(expanded_child)
18:    if all options for decision were considered then
19:        node.expanded := True
20:    return expanded_child

21: function SIMULATE(node)
22:    return estimated value of node

23: function BACKPROPAGATE(node, value)
24:    node.value + = value
25:    node.visits + = 1
26:    if node is not tree root then
27:        BACKPROPAGATE(node.parent, value)

28: function MCTS(decisions)
29:    Node tree_root
30:    tree_root.unresolved_decisions := decisions
31:    while insufficient number of simulations is performed do
32:        Node node := SELECT(tree_root)
33:        Node expanded_child := EXPAND(node)
34:        float value := SIMULATE(expanded_child)
35:        BACKPROPAGATE(expanded_child, value)
```

Algorithm 2 Procedure that allows us to make a decision based on a tree constructed using MCTS simulations.

```
function DECIDE(node, decision)
    best_child := the most visited child of node
    if node represents decision then
        return a result of decision in best_child
    else
        return DECIDE(child_node, decision)
```

B DERIVATION OF EQUATIONS FROM SECTION 3.2.1

$F(i)$ is a function computing number of nodes at the i-th level of the tree. k is a number of messages generated in a window. Only $r-1$ messages can be sent in one window. We number the tree starting with level 0 (i.e., in Figure 2 message m_1 is at level 0, m_2 at level 1, etc.). This means that level k represents the decision about k messages.

Then,

$$F(i) = \begin{cases} 2^i - \sum_{j=r}^{i} \binom{i}{j}, & \text{for } i \leq k \\ 2F(i-1) - \binom{k-1}{r-1}, & \text{for } i > k. \end{cases} \tag{1}$$

The case $i > k$ is a recursive function, so it is not too useful. Let us try to expand it:

$$\begin{aligned} F(i) &= 2F(i-1) - \binom{k-1}{r-1} = \\ &= 2\left(2F\left(i-2\right) - \binom{k-1}{r-1}\right) - \binom{k-1}{r-1} = \\ &= 2^2 F\left(i-2\right) - (2+1)\binom{k-1}{r-1} = \\ &= 2^{i-k}F(k) - \left(\sum_{j=0}^{i-k-1} 2^j\right)\binom{k-1}{r-1} = \\ &= 2^{i-k}F(k) - \left(2^{i-k}-1\right)\binom{k-1}{r-1} = \\ &= 2^{i-k}\left(2^k - \sum_{j=r}^{k}\binom{k}{j}\right) - \left(2^{i-k}-1\right)\binom{k-1}{r-1} = \\ &= 2^i - 2^{i-k}\sum_{j=r}^{k}\binom{k}{j} - \left(2^{i-k}-1\right)\binom{k-1}{r-1}. \end{aligned}$$

The result from above was given in the main text of this paper. Next, we stated that when $r \approx \frac{k}{2}$ the number of nodes is approximately 2^{i-1}. Because this is only an approximation, in order to simplify calculations, we consider only even k. The order of magnitude of the obtained result is not affected by this

assumption. We expand the result above for $r \approx \frac{k}{2}$:

$$
\begin{aligned}
F(i) &= 2^i - 2^{i-k} \sum_{j=r}^{k} \binom{k}{j} - \left(2^{i-k} - 1\right) \binom{k-1}{r-1} = \\
&= 2^i - 2^{i-k} \sum_{j=\frac{k}{2}}^{k} \binom{k}{j} - \left(2^{i-k} - 1\right) \binom{k-1}{r-1} = \\
&= 2^i - 2^{i-k} 2^{k-1} - \left(2^{i-k} - 1\right) \binom{k-1}{r-1} = \\
&= 2^{i-1} - \left(2^{i-k} - 1\right) \binom{k-1}{r-1} < 2^{i-1}
\end{aligned}
$$

Next we try to find out what will happen if the number of messages that can be sent is small compared to k:

$$
\begin{aligned}
F(i) &= 2^i - 2^{i-k} \sum_{j=r}^{k} \binom{k}{j} - \left(2^{i-k} - 1\right) \binom{k-1}{r-1} = \\
&= 2^i - 2^{i-k} \left(2^k - \sum_{j=0}^{r-1} \binom{k}{j}\right) - \left(2^{i-k} - 1\right) \binom{k-1}{r-1} = \\
&= 2^i - 2^i + 2^{i-k} \sum_{j=0}^{r-1} \binom{k}{j} - \left(2^{i-k} - 1\right) \binom{k-1}{r-1} = \\
&= 2^{i-k} \sum_{j=0}^{r-1} \binom{k}{j} - \left(2^{i-k} - 1\right) \binom{k-1}{r-1}
\end{aligned}
$$

by applying the binomial theorem we can limit it to:

$$
\begin{aligned}
F(i) &\leq 2^{i-k} (1+k)^{r-2} - \left(2^{i-k} - 1\right) \binom{k-1}{r-1} \\
&< 2^{i-k} (1+k)^{r-2}
\end{aligned}
$$

Hence, if the number of messages that can be sent tends to 0 (only a few messages can be sent), the number of nodes tends to being lower than 2^{i-k}.

REFERENCES

Best, Graeme, Michael Forrai, Ramgopal Mettu, and Robert Fitch (2018) “Planning-Aware Communication for Decentralised Multi-Robot Coordination”. In: *Proc. IEEE Int’l Conf. on Robotics and Automation (ICRA)* pp. 1050–1057.

Cazenave, Tristan, Abdallah Saffidine, and Nathan Sturtevant, eds. (2019) *Computer Games*. Springer.

Corera, Gordon (2016) “Google Achieves AI ’breakthrough’ at Go”. In: *BBC News*.

Coulom, Rémi (2006) “Efficient Selectivity and Backup Operators in Monte-Carlo Tree Search”. In: *Proc. Computers and Games*. Springer-Verlag.

Kocsis, Levente and Csaba Szepesvári (2006) “Bandit Based Monte-Carlo Planning”. In: *Machine Learning: ECML* ed. by Johannes Fürnkranz, Tobias Scheffer, and Myra Spiliopoulou. Lecture Notes in Computer Science. Berlin, Heidelberg: Springer, pp. 282–293.

Mansley, Chris, Ari Weinstein, and Michael L. Littman (2011) “Sample-Based Planning for Continuous Action Markov Decision Processes”. In: *Proc. Int’l Conf. on Automated Planning and Scheduling (ICAPS)* ICAPS’11. Freiburg, Germany: AAAI Press, pp. 335–338.

Pepels, Tom, Mark H. M. Winands, and Marc Lanctot (2014) “Real-Time Monte Carlo Tree Search in Ms Pac-Man”. In: *IEEE Trans. Comput. Intell. AI Games* 6.3, pp. 245–257.
